# Cost-effectiveness of Compression Therapy With Early Endovenous Ablation in Venous Ulceration for a Medicare Population

**DOI:** 10.1001/jamanetworkopen.2022.48152

**Published:** 2022-12-21

**Authors:** Hanke Zheng, Gregory A. Magee, Tze-Woei Tan, David G. Armstrong, William V. Padula

**Affiliations:** 1 Department of Pharmaceutical and Health Economics, School of Pharmacy, University of Southern California, Los Angeles; 2 Division of Vascular Surgery, Department of Surgery, Keck Medicine of University of Southern California, Los Angeles; 3 Southwestern Academic Limb Salvage Alliance (SALSA), Tucson, Arizona; 4 College of Medicine, Department of Surgery, University of Arizona, Tucson; 5 Leonard D. Schaeffer Center for Health Policy and Economics, University of Southern California, Los Angeles

## Abstract

**Importance:**

Venous leg ulcers (VLU) are the most common cause of lower extremity ulceration that commonly occur among older individuals and are characterized by a slow healing trajectory and frequent recurrence; in the United States, VLUs affect more than 600 000 people per year with an estimated cost of $3.5 billion. Clinical trial data show that early intervention with endovenous ablation substantially improves the healing rate and reduces recurrence among patients with VLUs, but there is a need to assess the cost-effectiveness of early endovenous ablation in the US context.

**Objectives:**

To evaluate the cost-effectiveness of early endovenous ablation of superficial venous reflux in patients with VLU from the US Medicare perspective.

**Design, Setting, and Participants:**

This economic evaluation used a Markov model to simulate the disease progression of VLU for patients receiving compression therapy with early vs deferred ablation over 3 years. The simulated cohort included patients with VLU aged 65 years and older who had clinical characteristics similar to those in the randomized Early Venous Reflux Ablation trial in the United Kingdom. Data were analyzed from September 2021 to June 2022.

**Main Outcomes and Measures:**

Direct medical costs, quality-adjusted life years (QALYs), and the incremental monetary benefits at a willingness-to-pay threshold of $100 000/QALY. Univariate and probabilistic sensitivity analyses were performed to test uncertainty of model results.

**Results:**

This model used a simulated cohort of patients with VLU aged 65 years and older enrolled in Medicare. Early ablation dominated, with a lower per-patient cost of $12 527 and an increase of 2.011 QALYs, whereas compression therapy with deferred ablation yielded a per-patient cost of $15 208 and 1.985 QALYs gained. At a $100 000/QALY cost-effectiveness threshold, the incremental net monetary benefit was $5226 per patient in favor of early ablation. Probability of healing, followed by the probability of recurrence, was the parameter with greatest impact on model uncertainty. The probabilistic sensitivity analysis showed that early ablation was cost-effective in 59.2% of simulations at the $100 000/QALY threshold.

**Conclusions and Relevance:**

In this economic evaluation of compression therapy with early endovenous ablation, early intervention was dominant, as it was cost saving and generated greater QALYs over 3 years from the US Medicare perspective. Payers should prioritize coverage for early ablation to prevent VLU complications rather than treat a costly outcome that also reduces patient well-being.

## Key Points

**Question:**

What is the cost-effectiveness of early intervention of endovenous ablation for patients with venous leg ulcerations (VLUs) from the Medicare perspective?

**Findings:**

In this economic evaluation, early ablation dominated, with a lower per patient cost of $12 527 and an increase of 2.011 quality-adjusted life years (QALYs), whereas compression therapy with deferred ablation yielded a per patient cost of $15 208 and 1.985 QALYs gained from the Medicare perspective over a 3-year time horizon.

**Meaning:**

In this study, compression therapy with early endovenous ablation was the dominant strategy, as it was cost saving and generated more QALYs over a 3-year time horizon from the US Medicare perspective.

## Teaser

This economic evaluation examines the cost-effectiveness of early endovenous ablation of superficial venous reflux in patients with venous leg ulcers from the US Medicare perspective.

## Introduction

Venous leg ulcers (VLU) are the most common cause of lower extremity ulceration and are characterized by slow healing trajectory and frequent recurrence. VLUs lead to significant disability, reduced quality of life, and tremendous economic burden.^1,2^ The United States, prevalence of VLU ranges from 0.15% to 0.30%, equating to approximately 600 000 cases per year, and is higher among women and older patients.^3,4^ The estimated annual cost of VLU treatment exceeds $3.5 billion.^5^

The traditional standard of care for VLU is compression therapy, which has been demonstrated as clinically beneficial.^6^ However, adherence to compression therapy is poor due to uncomfortableness.^7,8^ Alternative treatments including surgical interventions have been proposed. In addition to superficial venous surgery, minimally invasive endothermal treatments, including endovenous laser ablation, radiofrequency ablation, and mechanochemical ablation, have shown valid effectiveness in healing VLU.^9^ In a recent study, cyanoacrylate adhesive ablation was found to be cost-effective compared with surgical stripping for treating varicose veins from the societal perspective in Spain, considering the opportunity costs of medical leave.^10^

The Early Venous Reflux Ablation (EVRA) trial conducted in the UK suggests that early ablation with compression therapy substantially increases the healing rate and reduces the chance of recurrence of VLU.^11,12^ Early ablation has also shown to be cost-effective in the long term from the UK health care sector perspective.^12^ Given the uniqueness and complexity of the US health care system, particularly in the Medicare and dual-eligible (ie, Medicare and Medicaid) populations, we aim to assess the cost-effectiveness of early endovenous ablation with compression therapy among older patients with VLU from the US Medicare perspective. These economic data can be used by payers that participate in Medicare programs to cover and rank early ablation for VLU with respect to other alternative forms of treatment.

## Methods

### Model Overview

Our study analyzed the cost-effectiveness of compression therapy with early vs deferred endovenous ablation among VLU patients aged 65 years and older from the US Medicare perspective, following methods prescribed by the US Panel on Cost-effectiveness in Health and Medicine and the Consolidated Health Economic Evaluation Reporting Standards (CHEERS) reporting guidelines.^13,14^ Per the Common Rule, this study was exempted from institutional review board approval and informed consent because no human participants were involved.

The treatment assignment and the clinical features of the patients included in the model were based on the UK EVRA trial.^11,12^ Specifically, the early intervention was defined as receiving compression therapy and undergoing early endovenous ablation, ie, performed within 2 weeks after VLU became clinically significant. Patients receiving deferred intervention would receive compression therapy alone and deferred the ablation until the ulcer had healed or after 6 months if the ulcer had not healed.

**Figure 1.**
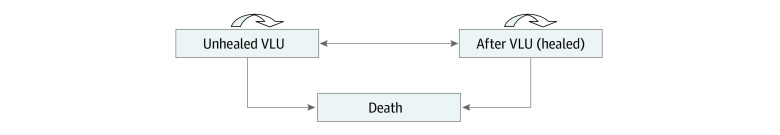
Markov Model Simulation of Venous Leg Ulceration (VLU) Disease Progression

Patients entered the model with an open VLU for a period of between 6 weeks and 6 months, an ankle-brachial index of 0.8 or higher, and primary or recurrent superficial venous reflux that was deemed by the treating clinician to be clinically significant. We developed a Markov model with 3 mutually exclusive health states (ie, unhealed VLU, post-VLU [healed], and death) to simulate the disease progression of VLU (Figure 1). Patients began in the unhealed VLU state and could stay unhealed or transition to post-VLU (healed) or death states based on their assigned transition probabilities.

Monthly cycles were used to assess the costs and outcomes associated with the 3 health states. The time horizon used for the base case was 3 years. Both costs and health outcomes were discounted at an annual rate of 3%. All monetary terms were converted to 2021 US dollars using the Medical Component of the Consumer Price Index.^15^ The primary outcomes of the model included the costs associated with VLU treatment and management and quality-adjusted life years (QALYs) gained per patient. These data were used to derive the incremental net monetary benefits (NMB) at a cost-effectiveness threshold of $100 000/QALY.

### Probabilities

Transition probabilities between unhealed VLU and post-VLU (healed) were calculated based on the healing rate and recurrence rate from the EVRA trial (Table 1).^11,12,16^ The EVRA trial reported the healing rate at 6 months and 12 months as well as the recurrence rate from 1 year up to 3 years after the treatment initiation. Most patients were healed within 6 months (85.6% in the early ablation group vs 76.3% in the deferred ablation group); more were healed within 12 months (93.7% in the early ablation group vs 85.8% in the deferred ablation group). The recurrence rates at 3 years were 24.5% for the early ablation group and 29.9% for the deferred group. Based on the EVRA trail data, we calculated the monthly between-state transition probabilities applying the declining exponential approximation of life expectancy methods.^17^ The probability of healing after 12 months was assumed to be half of that between 6 and 12 months to reflect the reality that some patients might have smaller chance to heal. Given the insufficient evidence for an increased risk of mortality associated with VLU, we used all-cause mortality for the general population age 65 years and older in the United States.^16^

**Table 1.** Transition Probability Base Case Inputs and Range for Sensitivity Analyses

Parameter	Cycle transition probability (±20% range)	Source
**Compression with early ablation**		
Probability of healing		
Month 1 to <6	0.133 (0.106-0.160)	Gohel et al,^12^ 2020
Month 6 to <12	0.090 (0.072-0.108)	Gohel et al,^12^ 2020
Month ≥12	0.045 (0.036-0.054)	Gohel et al,^12^ 2020
Probability of recurrence		
Month 1 to <12	0.011 (0.009-0.013)	Gohel et al,^12^ 2020
Month 12 to <24	0.004 (0.003-0.004)	Gohel et al,^12^ 2020
Month 24 to <36	0.007 (0.006-0.009)	Gohel et al,^12^ 2020
**Compression with deferred ablation**		
Probability of healing		
Month 1 to <6	0.119 (0.096-0.143)	Gohel et al,^12^ 2020
Month 6 to <12	0.065 (0.052-0.078)	Gohel et al,^12^ 2020
Month ≥12	0.032 (0.026-0.039)	Gohel et al,^12^ 2020
Probability of recurrence		
Month 1 to <12	0.016 (0.013-0.019)	Gohel et al,^12^ 2020
Month 12 to <24	0.005 (0.004-0.006)	Gohel et al,^12^ 2020
Month 24 to <36	0.007 (0.005-0.008)	Gohel et al,^12^ 2020
All-cause mortality		
Month 1 to <12	0.001 (0.0008-0.0012)	CDC,^16^ 2017
Month 12 to <24	0.001 (0.0008-0.0012)	CDC,^16^ 2017
Month 24 to <36	0.001 (0.0008-0.0012)	CDC,^16^ 2017

Abbreviation: CDC, Centers for Disease Control and Prevention.

### Costs

Direct medical costs associated with VLU treatment were considered in the model (Table 2).^18,19,20,21^ Specific cost components included costs of endovenous ablation, compression therapy, pain medication, additional home health, and hospitalization due to infections and complications of VLU. The Medicare national average reimbursement rates in accordance with the *Current Procedural Terminology* (*CPT*) codes and diagnosis-related groups (DRGs) identified for the VLU-related medical procedures and services were sourced from the Centers for Medicare & Medicaid Services (CMS) data sets and published literature.^18,19,20,21^ We derived the total costs as the product of quantity used and the relevant unit costs. The *CPT* codes were identified to target costs for the endovenous ablation (mechanochemical ablation: 36473 and 36474; endovenous radiofrequency: 36475 and 36476; endovenous laser: 36478 and 36479).^22^ In the EVRA trial, the endovenous treatment decision was left to the discretion of the clinicians, and no granular data about the specific procedure utilization were reported. Thus, according to the expert opinion of Gregory A. Magee, MD, MSc (May 10, 2021; online meeting), we assumed 40% of patients to be treated with mechanochemical ablation, 40% to receive endovenous radiofrequency, and 20% to receive endovenous laser. Among them, 10% were assumed to have more than 1 vein treated on a single extremity. Since the Medicare reimbursement rate for these 3 ablation procedures were nearly equivalent (Table 2), the assumption about specific procedure utilization was not expected to result in great variations in the calculated costs.

**Table 2.** Direct Costs of Venous Leg Ulceration Treatment

Cost parameter	*CPT* or DRG code	Medicare costs (±20% range), $	Source
Intervention costs			
Endovenous radiofrequency	36475	1323 (1059-1588)	CMS,^18^ 2021
Radiofrequency added on with multiple veins treatment	36476	314 (251-377)	CMS,^18^ 2021
Endovenous laser	36478	1215 (972-1458)	CMS,^18^ 2021
Laser added on with multiple veins treatment	36479	138 (111-166)	CMS,^18^ 2021
Mechanochemical			
Ablation	36473	1448 (1158-1737)	CMS,^18^ 2021
Ablation added on with multiple veins treatment	36474	296 (237-356)	CMS,^18^ 2021
Physician payment, facility			
Physician initial visit			
Evaluation	99203	85 (68-102)	CMS,^18^ 2021
Debridement	11042	63 (51-76)	CMS,^18^ 2021
Physician, debridement, established visit	97597	36 (29-44)	CMS,^18^ 2021
Physician, compression only	99212	36 (29-44)	CMS,^18^ 2021
Facility reimbursement			
Initial visit	99213	86 (69-104)	Nherera et al,^19^ 2016
Debridement, initial visit	11042	220 (176-264)	Nherera et al,^19^ 2016
Debridement established visit	97597	114 (91-137)	Nherera et al,^19^ 2016
Compression only	29581	83 (66-99)	Nherera et al,^19^ 2016
Home health			
Home health (60-d episode)	C2F2S1	2808 (2246-3370)	Carter et al,^20^ 2014
Compression			
Compression stocking (per pair for 6 mo)	A6532	72 (58-86)	Carter et al,^20^ 2014
Hospitalization costs			
Skin debridement with complication	571	10 832 (8665-12 998)	CMS,^21^ 2019
Skin ulcer with complication	593	8882 (7105-10 658)	CMS,^21^ 2019
Cellulitis			
No major complication	603	5562 (4449-6674)	CMS,^21^ 2019
Major complication	602	9872 (7898-11 847)	CMS,^21^ 2019
Pain medications (prescription drugs), calculated monthly cost			
Amitriptyline	NA	43 (35-52)	Carter et al,^20^ 2014
Gabapentin	NA	124 (99-149)	Carter et al,^20^ 2014
Hydrocodone	NA	22 (17-26)	Carter et al,^20^ 2014

Abbreviations: CMS, Centers for Medicare & Medicaid Services; *CPT*, *Current Procedural Terminology*; DRG, diagnosis-related group; NA, not applicable.

Costs of compression stocking and outpatient visits associated with debridement and compression therapy were also considered. For the outpatient visits, we assumed 1 visit per week to reflect a typical frequency of visits until the wound healed.^20^ According to the CMS reimbursement policy, no compression billing is allowed if debridement occurs.^20^ Taking the established data from the existing cost-effectiveness analyses, the estimated likelihood of debridement per week was 12.5%, and it was assumed to only occur within the first 3 months, representing a reasonable debridement frequency of the VLU treatment.^20,23^ After that, only *CPT* codes for an established clinic visit for compression were used to generate costs for outpatient visits. Additionally, 25% of patients would have 1 home health care visit per week to change dressing.^20^ We used the code C2F2S1 from the Medicare home health prospective payment system to obtain the cost of home health within a 60-day episode for Medicare.

We incorporated the costs of hospitalization due to VLU and common infections, such as cellulitis, into the model.^24,25,26^ For patients with unhealed VLU, the annual rate of hospitalization for VLU visits and infections were referenced from a cost-effectiveness analysis assessing the management of chronic VLU, which translated to a monthly probability of 0.83% for hospitalization due to skin debridement with complications and/or due to skin ulcer with complications, 0.08% for cellulitis with major complications, and 0.33% for cellulitis without complications. The DRG codes for VLU were extracted to derive the hospitalization costs, including DRG 571 (skin debridement with complications and comorbidities) and 593 (skin ulcer with complications and comorbidities).^19,20^ DRGs 602 and 603 (cellulitis with and without major complications) were used to determine the hospitalization costs for infections.^19,20^ The national average Medicare hospitalization payment was referenced to calculate the costs to the payer.^21^ We omitted the costs associated with antibiotics for infection because it was low and negligible.^20^

Given the fact that patients with VLU commonly exhibit pain caused by the disease, our study considered the costs of pain management directly associated with VLU, including amitriptyline (40%), gabapentin (10%), and hydrocodone (50%).^20,27^ For patients healed from VLU, we included the costs of compression stockings as it is considered as a standard care in the management after VLU.^28^

### Health Utilities

The utilities measuring patients’ quality of life (QOL) were assigned by health state, based on data reported from the Euro-QOL 5-Domain index of US nationally representative QALYs reported by Sullivan and Ghushchyan.^29^ We took the utility score of people affected by chronic ulcer of skin, based on *International Classification of Diseases, Ninth Revision *(*ICD-9*) codes 707.0 for the unhealed VLU state (0.69) (eTable in Supplement 1). The utility score for the healed VLU state (0.75) was derived from another study^30^ that assessed the effect of VLU on QOL in the UK population (eTable in Supplement 1). To account for the impact of aging on people’s preference of QOL, we adjusted these utility scores to estimate the perception of individuals aged between 65 and 74 years using the US population disutility for aging.^31^

### Sensitivity Analyses

To understand the how changes in value of a specific parameter would affect the model results, univariate sensitivity analysis was performed by varying each parameter within its ±20% range. Probabilistic sensitivity analyses (PSAs) were carried out to test concurrent uncertainty of the base case results based on model structure, parameter sourcing, and sampling simultaneously. We generated 10 000 Monte Carlo simulations for the PSA by varying all model inputs according to their given distributions. We then created a cost-effectiveness acceptability curve using the simulated cases, which allows for the visualization of the likelihood that early ablation is cost-effective at varying willingness-to-pay thresholds. The expected value of perfect information (EVPI) per person was calculated to inform the amount to invest for future research to eliminate the uncertainty for the recommended optimal strategy.

### Budget Impact Analysis

To estimate the monetary impact of implementing early ablation for patients with VLU from the payer’s perspective, we conducted a budget impact analysis in a hypothetical population of 1 million members, assuming 1000 VLU cases among the members having clinical characteristics as presented in the model. We reported the total budget impact of early endovenous ablation and the per-member-per-month (PMPM) amount at 1 year, 3 years, and 5 years after the intervention.

### Statistical Analysis

Descriptive statistics of costs and QALYs over 1 year and 3 years are presented. Excel 2016 (Microsoft Corp) was used for all statistical calculations, simulations, and figure production. Data were analyzed from September 2021 to June 2022.

## Results

We created a simulated cohort of patients with VLU aged 65 years and older enrolled in Medicare. Compression therapy with early endovenous ablation was the dominant option by yielding more QALYs at a lower cost. The total cost of early intervention was $12 527, and the total QALYs gained were 2.011 per person at a 3-year time horizon from the US Medicare perspective (Table 3). In contrast, compression therapy with deferred ablation yielded a total cost of $15 208 and 1.985 QALYs per person. Thus, at the cost-effectiveness threshold of $100 000/QALY, the incremental NMB of early ablation was $5226 per person at 3 years. The base case results of early ablation were dominant at both 1 year and 3 years, with an increasing incremental NMB with longer time horizon.

**Table 3.** Base Case Results

Treatment	$		QALYs	Incremental QALYs	ICER	INMB, $
	Cost	Cost difference				
1 y						
Deferred ablation	8423	−636	0.699	0.004	Early ablation dominates	981
Early ablation	7787		0.703			
3 y						
Deferred ablation	15 208	−2681	1.985	0.026	Early ablation dominates	5226
Early ablation	12 527		2.011			

Abbreviations: ICER, incremental cost-effectiveness ratio; INMB, incremental net monetary benefits; QALYs, quality-adjusted life years.

### Sensitivity Analyses

In the univariate sensitivity analysis, the parameter showing the greatest impact on the incremental NMB was the probability of healing, followed by the probability of recurrence. If varying the probability of healing for patients receiving early ablation in the first 6 months within its ±20% range, the incremental NMB would range from −$17 192 to $27 691 per patient.

**Figure 2.**
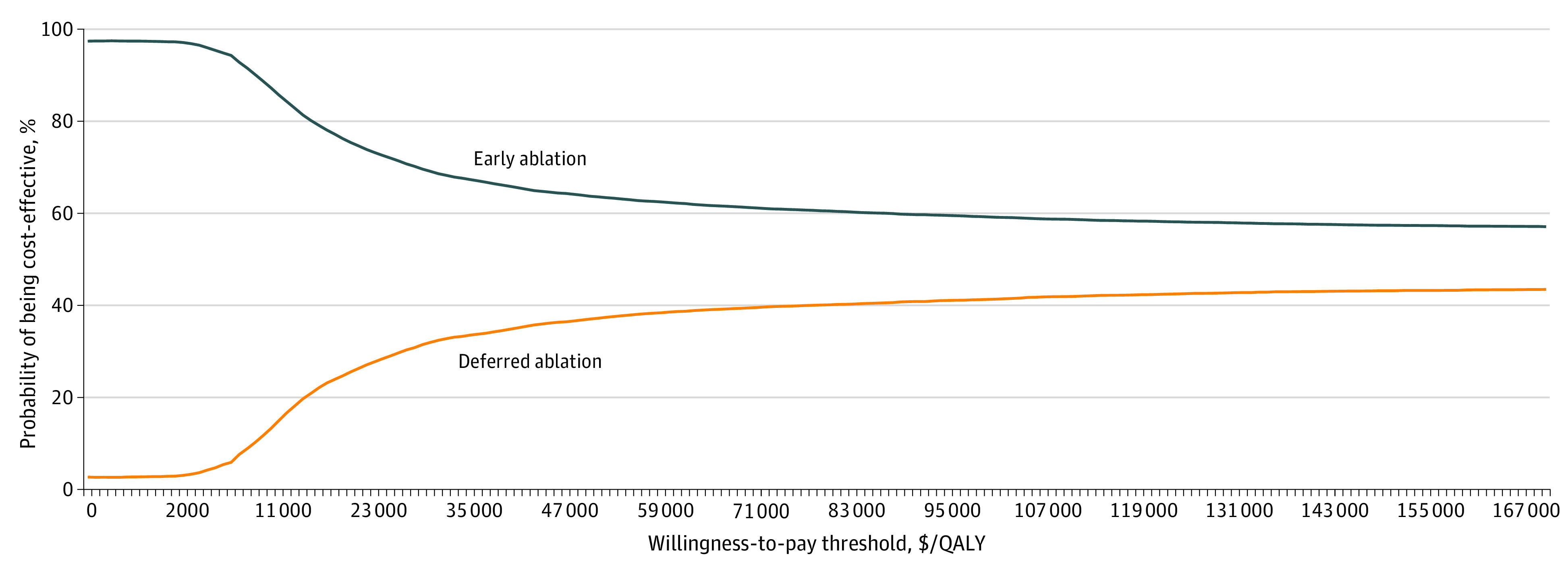
Cost-effectiveness Acceptability Curve of Early vs Deferred Ablation Abbreviation: QALY, quality-adjusted life years.

Based on the 100 000 cases simulated in the PSA, the average incremental NMB generated by early intervention was $5286 per person. The cost-effectiveness acceptability curve derived from the PSA illustrates a greater likelihood of early ablation being cost-effective regardless of the cost-effectiveness threshold (Figure 2). At $100 000/QALY thresholds, compression with early endovenous ablation was cost-effective in 59.2% of the 100 000 simulated cases, and this probability decreased to 57.4% if applying a $150 000/QALY threshold. At a $100 000/QALY threshold, the EVPI was $6341 per person from the Medicare perspective, meaning that investing $6341 in research on each person with VLU would increase our confidence in the recommendation of optimal strategy.

### Budget Impact Analysis

Assuming 1000 patients with VLU in a hypothetical 1-million-member health plan, compression therapy with early endovenous ablation generated a total cost saving of $636 238 at 1 year and $2 680 246 at 3 years, which was equivalent to a PMPM difference of $0.053 at 1 year and $0.075 at 3 years. Therefore, early endovenous ablation for patients with VLU was cost-saving from a payer’s perspective,

## Discussion

Our study addressed the unmet need for an economic evaluation of early intervention of endovenous ablation for patients with VLU. According to our analysis, compression therapy with early endovenous ablation dominated deferred ablation since it provided care for VLU at a lower cost and improved patient QOL over a 3-year period from the Medicare perspective. This finding is robust, as supported by PSA results. These cost-effectiveness results apply specifically to a simulated cohort of individuals aged 65 years and older, among whom the highest concentration of VLUs occur in the United States.^3,4^ Payers wishing to deliver greater health benefits for their patients while saving costs should place early endovenous ablation at the top of their formulary for patients with VLU. While the calculated budget saving is insignificant, it increases the likelihood for payers to adopt early ablation without displacing resources for other patients in need.

Medicare programs stand to benefit from the findings of the EVRA trial combined with this economic evaluation. Medicare spends more than $1.0 billion per year on the chronic management of VLUs.^32^ The findings of this study suggest that this spending could be conserved if more interventional procedures, such as early endovenous ablation, are undertaken decisively for patients with clinically significant VLU to mitigate downstream costs of chronic would care, not to mention gains in clinical benefits for patients through intact skin.

Health systems that implement these types of early interventions need to be properly incentivized by Medicare payments and performance measures to be sustainable. Currently, health systems reap approximately $100 billion per year in chronic wound management because conditions including VLU, pressure injury, diabetic foot ulcer, and others require chronic wound care in outpatient and long-term care settings.^32,33^ Health systems that transition to measures such as early endovenous ablation may lose money by providing procedures up front for a fraction of the cost to avert downstream chronic wound care costs. As a result, Medicare should work with health systems to increase the reimbursement rate for early endovenous ablation while maintaining its dominance as cost-effective to increase incentives for its use and to compensate for losses from less chronic wound care in the long run. Medicare could also develop a pay-for-performance incentive that would reward health systems that improve population health by effectively managing the prevention of escalated VLU cases in the chronic phase to leverage value of care, as has been piloted with other types of health outcomes, including cardiovascular disease, diabetes, and cancer.^34,35,36,37^

### Limitations

This study has several limitations. First, data on the clinical efficacy of early endovenous ablation are based on a trial from the United Kingdom and do not necessarily represent outcomes that pertain to a US patient population. For example, in the EVRA trial, the average age of the participants randomized with early intervention was 67.0 years, and it was 68.9 years among those assigned to deferred intervention. The US Medicare population is likely to be older than the trial population, as most Medicare beneficiaries are aged 65 years and older.^38^ Second, the EVRA trial examined the clinical benefits of early ablation compared with delayed ablation with compression therapy in a controlled setting. Since we know compression therapy has low adherence, the results of this economic evaluation represent a lower-bound given that patients in real-world settings gain fewer clinical benefits from delayed ablation with compression therapy when they lack adherence. Third, in the EVRA trial, the type of ablation technology to apply was left to the discretion of the clinical team. Due to the lack of data, an assumption was made about the share of different ablation procedures to account for the ablation-related costs in the US context based on expert opinion, but this assumption was not expected to result in great variation of the results because the costs of these ablation procedures borne by Medicare are close (Table 2). Furthermore, the healing rate after 12 months was assumed to be half of that between months 6 and 12 because it was not reported in the EVRA data. We captured the uncertainty of the assumption in our sensitivity analyses. In addition, the economic model did not control for variability in the population of patients with VLU, of which there are many sociodemographic causes. Patients in rural areas, or patients who are predisposed to health disparities that perpetuate challenges to accessing specialty care for VLU, may have less predictable outcomes.^33,39^ However, one could make that argument that a swift and effective treatment such as early ablation would be more efficient, particularly for individuals facing health disparities, than treatments that require consistent follow-up in the long run for chronic wound management.

## Conclusions

In this economic evaluation of compression therapy with early endovenous ablation, we found early ablation to be a cost-effective alternative to delayed ablation with compression therapy for Medicare patients diagnosed with VLU. Medicare should consider innovative payment models that increase incentives for health systems to deploy early endovenous ablation to all eligible patients with VLU. Doing so will save on the excessive costs of chronic wound care and improve clinical benefits for patients that currently face long durations of follow-up and extreme pain caused by VLU.

## Notes

Supplement 1. **eTable. **Utility Values of Venous Leg Ulceration
eReferences.

Supplement 2.
Data Sharing Statement
