# Supplementary material for: Cost-effectiveness of Compression Therapy With Early Endovenous Ablation in Venous Ulceration for a Medicare Population
Source: JAMA Netw Open. 2022 Dec 21;5(12):e2248152. doi: 10.1001/jamanetworkopen.2022.48152 (PMC9857339; doi:10.1001/jamanetworkopen.2022.48152)
Supplement: Supplement 1. — eTable. Utility Values of Venous Leg Ulceration eReferences. [file jamanetwopen-e2248152-s001.pdf]

Supplemental Online Content

Zheng H, Magee GA, Tan TW, Armstrong DG, Padula WV. Cost-effectiveness of compression therapy with early endovenous ablation in venous ulceration for a Medicare population. *JAMA Netw Open.* 2022;5(12):e2248152. doi:10.1001/jamanetworkopen.2022.48152

eTable. Utility Values of Venous Leg Ulceration

eReferences.

This supplemental material has been provided by the authors to give readers additional information about their work.

© 2022 Zheng H et al. *JAMA Network Open.*

eTable. Utility Values of Venous Leg Ulceration

State	Utility (± 20% range)	Reference
Unhealed VLU	0.69 (0.55-0.82)	Sullivan, [1] 2006; Jiang, [2] 2021.
Healed ulcer	0.75 (0.69-0.90)	Jiang, [2] 2021; Lglesias, [3] 2005.
Death	0	

VLU, venous leg ulcer.

eReferences.

1. Sullivan PW, Ghushchyan V. Preference-Based EQ-5D index scores for chronic conditions in the United States. *Med Decis Making.* 2006;26(4):410-420.

2. Jiang R, Janssen MFB, Pickard AS. US population norms for the EQ-5D-5L and comparison of norms from face-to-face and online samples. Qual Life Res. 2021;30(3):803-816.

3. Iglesias CP, Birks Y, Nelson EA, Scanlon E, Cullum NA. Quality of life of people with venous leg ulcers: a comparison of the discriminative and responsive characteristics of two generic and a disease specific instruments. *Qual Life Res.* 2005;14(7):1705-1718.

© 2022 Zheng H et al. *JAMA Network Open.*
