# Supplementary material for: Cost-effectiveness of Compression Therapy With Early Endovenous Ablation in Venous Ulceration for a Medicare Population
Source: JAMA Netw Open. 2022 Dec 21;5(12):e2248152. doi: 10.1001/jamanetworkopen.2022.48152 (PMC9857339; doi:10.1001/jamanetworkopen.2022.48152)
Supplement: Supplement 2. — Data Sharing Statement [file jamanetwopen-e2248152-s002.pdf]

Data Sharing Statement

Zheng. Cost-effectiveness of Compression Therapy With Early Endovenous Ablation in Venous Ulceration for a Medicare Population. *JAMA Netw Open*. Published December 21, 2022. doi:10.1001/jamanetworkopen.2022.48152

Data
Data available: Yes
Data types: Other (please specify)
Additional Information: All the data used in this study were sourced from published literature and the sources have been provided in the manuscript.
How to access data: All the data used in this study were sourced from published literature and the sources have been provided in the manuscript.
When available: With publication

Supporting Documents
Document types: None

Additional Information
Who can access the data: Anyone requesting the data
Types of analyses: Economic evaluation
Mechanisms of data availability: After approval of a proposal
